# Molecular and morphological description of a novel microsporidian *Inodosporus fujiokai* n. sp. infecting both salmonid fish and freshwater prawns

**DOI:** 10.1017/S003118202200141X

**Published:** 2023-01

**Authors:** Tetsuya Yanagida, Nanami Asai, Michitaka Yamamoto, Kazuhiro Sugahara, Takuto Fujiwara, Sho Shirakashi, Hiroshi Yokoyama

**Affiliations:** 1 Laboratory of Parasitology, Joint Faculty of Veterinary Medicine, Yamaguchi University, Yamaguchi 753-8511, Japan; 2 Aquaculture Research Institute, Kindai University, Wakayama 649-2211, Japan; 3 Shiga Prefecture Fisheries Management Division, Otsu, Shiga 520-8577, Japan; 4 Shiga Prefectural Fisheries Experiment Station, Shiga 522-0057, Japan; 5 Faculty of Veterinary Medicine, Okayama University of Science, Ehime 794-8555, Japan

**Keywords:** Lake Biwa, microsporidia, molecular phylogeny, polymorphic life cycle, ultrastructure

## Abstract

A new microsporidian disease of cultured rainbow trout *Oncorhynchus mykiss* has recently been confirmed in Japan, and the causative species was tentatively designated as *Microsporidium* sp. RBT-2021. Involvement of common prawn *Palaemon paucidens* in its transmission was suggested based on the previous feeding trials, although the microsporidian infection in *P. paucidens* was not confirmed. In this study, *P. paucidens* in Lake Biwa, Japan was investigated for microsporidian infection and 4 types of spores (types 1–4) were newly found. The nucleotide sequence of the small subunit ribosomal RNA gene was identical between type 1 and *Microsporidium* sp. RBT-2021, indicating they are conspecific. However, intriguingly, the spore morphology and the mode of development in fish and prawn were strikingly different. Morphological observations revealed type 1 in the prawn possesses characteristics of the genus *Inodosporus* Overstreet and Weidner, 1974, while *Microsporidium* sp. RBT-2021 in the trout exhibited the characteristics of the genus *Kabatana* Lom, Dyková and Tonguthai, 2000. In the phylogeny, type 1 was placed within a clade comprising *Kabatana* spp. and *Inodosporus octosporus*. Based on the morphological and molecular analyses, we describe *Microsporidium* sp. RBT-2021 as *Inodosporus fujiokai* n. sp. Together with the success of the previous prawn-feeding trials, this study strongly suggests *I. fujiokai* n. sp. has a multi-host life cycle utilizing fish and crustacean hosts and different modes of development in each host. Such polymorphic life cycle has barely been known among fish microsporidians. This study also suggests that the genus *Kabatana* is a junior synonym of the genus *Inodosporus*.

## Graphical abstract

**Figure:**
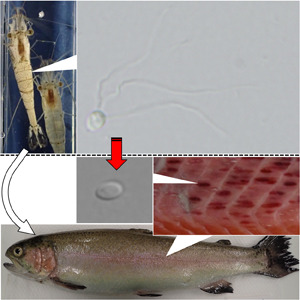

## Introduction

Microsporidia are a group of eukaryotic intracellular obligate parasites infecting a wide range of invertebrate and vertebrate organisms. Recent molecular phylogenetic studies indicate that microsporidia are closely related to fungi (James *et al*., 2006; Capella-Gutiérrez *et al*., 2012). Microsporidia form tiny unicellular spores ranging in size from 1 to 20 *μ*m, but mostly about 1–5 *μ*m (Cali *et al*., 2016). The life cycle of microsporidians consists of (a) a proliferative phase (merogony), (b) the spore formation phase (sporogony) and (c) environmental/infective phase (mature spore) (Cali *et al*., 2016). While the life cycle can be completed in a single host, some species are demonstrated to have dimorphic or polymorphic life cycles involving multiple hosts (Vávra and Larsson, 2014). To date, approximately 1500 microsporidian species have been described (Stentiford *et al*., 2013), and more than 160 species belonging to 21 genera have been reported from fish hosts (Mansour *et al*., 2020). Close attention has been paid to some fish microsporidians because of their negative effects on the fisheries and aquaculture industries worldwide. For example, *Loma salmonae*, the causative agent of microsporidial gill disease, infects the gills of salmonids and causes mortality in the net-pen salmon farming industry, especially in the Pacific coast of North America (Shaw *et al*., 2000; Speare and Lovy, 2012). *Pleistophora hyphessobryconis*, the cause of neon tetra disease, is one of the most deleterious microsporidians in freshwater ornamental fish with a broad host range (Li *et al*., 2012; Winters *et al*., 2015). Infection of *P. hyphessobryconis* in the zebrafish *Danio rerio*, an important model animal, has been confirmed in research facilities (Sanders *et al*., 2010), and thus this microsporidian is listed as a target pathogen in the health monitoring programme of zebrafish. In Japan, *Microsporidium seriolae* has been causing great economic losses to amberjack farming, mainly because of the unsightly cysts in the somatic muscle of the infected fish (Yokoyama, 2017; Yanagi *et al*., 2021).

In spite of its importance in the fisheries, the general biology and life cycle of fish microsporidians are mostly unknown. Among the above-mentioned species, fish-to-fish direct transmission has been demonstrated for *L. salmonae* (Shaw *et al*., 1998, 2000) and *P. hyphessobryconis* (Sanders *et al*., 2010). On the other hand, horizontal transmission between fish does not seem to occur in *M. seriolae*, indicating the requirement of unknown intermediate or alternate host(s) in its life cycle (Yokoyama *et al*., 2011). The involvement of intermediate or alternate host(s) has also been suggested for *Kabatana takedai*, which infects the trunk muscle and heart of salmonid fish, but its transmission route has not yet been unveiled (Fujiyama *et al*., 2002). The lack of the knowledge on the life cycle hampers the control of microsporidian diseases in fisheries and aquaculture. Recently, Stentiford *et al*. (2018) suggested the trophic transfer of a shrimp microsporidian *Inodosporus octosporus* (syn. *Inodosporus octospora*) between crustacean and fish hosts based on the similarity in the small subunit ribosomal RNA gene (SSU rDNA) sequence with *Kabatana* sp. JI-2008, which was described from the 2-spotted goby *Gobiusculus flavescens* in Sweden (Barber *et al*., 2009). Stentiford *et al*. (2018) further suggested the synonymy of the genera *Inodosporus* (shrimp microsporidians) and *Kabatana* (fish microsporidians), although the conclusion was not made.

Our previous study also indicated the involvement of a crustacean host in the transmission of a fish microsporidian newly found from the cultured rainbow trout *Oncorhynchus mykiss* in Japan (Yamamoto *et al*., 2021). The rainbow trout with a history of being fed wild-caught common prawn *Palaemon paucidens* exhibited the petechiae-like spots on the trunk muscle and microsporidian spores were detected from the affected area. The light microscopical observation and molecular analysis strongly suggested that the microsporidian is an undescribed species closely related to *K. takedai*. The microsporidian was tentatively named *Microsporidium* sp. RBT-2021, the generic name for uncertain generic position, due to the lack of ultrastructural analysis (Sprague, 1977). Our prawn-feeding trials using *O. mykiss* successfully reproduced the microsporidian infection, indicating that *P. paucidens* plays an important role in the transmission of *Microsporidium* sp. RBT-2021. However, microsporidian infection in the prawns was not confirmed and thus the life cycle of *Microsporidium* sp. RBT-2021 remained to be elucidated (Yamamoto *et al*., 2021).

In this study, microsporidian infection in wild *P. paucidens* was investigated to demonstrate its involvement in the life cycle of *Microsporidium* sp. RBT-2021. Furthermore, ultrastructural observation and molecular analysis were conducted to identify the taxonomic position of *Microsporidium* sp. RBT-2021.

## Materials and methods

### Sample collection

Prawns (*P. paucidens*) were collected from Lake Biwa, Shiga Prefecture, Japan at Shiga Prefectural Fisheries Experiment Station (35°15′48″N, 136°12′50″E) using a dip net in May 2019 and June 2020. In 2019, approximately 4900 prawns (average body weight 0.28 g, body length 37.5 mm) were captured in 1 day (30th May). Of those, individuals showing opaque and whitish tissues under carapace were distinguished from others. These opaque prawns as well as haphazardly selected 100 individuals of apparently normal (non-opaque) prawns were subjected to microsporidian examination. In 2020, hundreds to thousands of prawns were captured daily and opaque individuals were separated to check for microsporidian infections.

The prawns were dissected, and a small portion of the abdominal muscle was observed by wet mount using light microscopy. When microsporidian spores were detected, remaining tissue of the infected prawn was frozen and/or fixed with 70% ethanol for further analyses. Some of the infected prawns were chosen for histopathological and/or transmission electron microscope (TEM) observations and their tissues were fixed accordingly. To compare the ultrastructure of the developmental stages and spores of microsporidia from *P. paucidens* with those of *Microsporidium* sp. RBT-2021, muscle tissue of the infected *O. mykiss* was also prepared for TEM observation.

In this study, *M. seriolae* was also used for molecular phylogeny. Despite the lack of detailed ultrastructural observation for *M. seriolae*, it has sometimes placed in the genus *Kabatana* (Lom *et al*., 1999, 2001; McGourty *et al*., 2007; Barber *et al*., 2009; Casal *et al*., 2010). However, the phylogenetic position of *M. seriolae* has been unstable because its SSU rDNA sequences available in the database are relatively short. We thus tried to obtain a longer SSU rDNA sequence of *M. seriolae* to evaluate its phylogenetic position. The spores of *M. seriolae* used for this study were obtained from a dead juvenile *Seriola dumerili* farmed in Ehime Prefecture, Japan.

### Light microscopy of spores and histology

For the light microscopic observations of wet mount spores, fresh or frozen samples were used. Spores were photographed at 1000× magnification and measured (*n* = 20 or 40) using image analysis software ImageJ (https://imagej.nih.gov/ij/).

For the histological observation, the muscle tissues of the infected prawns were fixed with Davidson's fixative and then processed for paraffin embedding. Sections were cut at 4 *μ*m, deparaffinized with a graded ethanol series, rehydrated to tap water and stained with haematoxylin and eosin (H&E). Stained sections were dehydrated with a graded ethanol series before mounted by EUKITT^®^ mounting medium (Orsatec, Germany).

### Molecular analysis

Parasite DNA was extracted from frozen or ethanol-fixed specimens using a DNA Mini Kit (Qiagen, Hilden, Germany) following the manufacturer's protocol. For the amplification of rDNA, a set of V1f (5′-CAC CAG GTT GAT TCT GCC-3′) and 580r (5′-GGT CCG TGT TTC AAG ACG G-3′) or V1f and 1492r (5′-GGT TAC CTT GTT ACG ACT T-3′) primers were used (Vossbrinck *et al*., 1993; Nilsen, 2000). Polymerase chain reaction (PCR) was carried out in 20 *μ*L reaction volumes containing 15 pmol of each primer, 0.5 units of ExTaq Hot Start version (TaKaRa Bio Inc., Shiga, Japan), 0.2 mm of dNTP, 1× ExTaq buffer with a final MgCl_2_ concentration of 2.0 mm and 1.0 *μ*L of genomic DNA. PCR amplification consisted of initial denaturation of 94°C for 2 min, 40 cycles of 94°C for 15 s, 55°C for 15 s and 72°C for 30 s, and a final extension at 72°C for 1 min. PCR products were treated with ExoSAP-IT (Thermo Fisher Scientific, Waltham, USA) to remove excess primers and dNTPs, and subsequently sequenced directly using a BigDye Terminator v3.1 and 3130 DNA sequencer (Thermo Fisher Scientific, Waltham, USA). In this study, the nucleotide sequence of rDNA was determined for each type of microsporidians from the prawns and *M. seriolae* from the greater amberjack.

The sequences obtained by direct sequencing were compared by a BLAST search against the sequences available in the International Sequence Database Collaboration (INSDC) database. Then, the sequences were aligned by MAFFT (Katoh *et al*., 2002; Katoh and Standley, 2013) with the rDNA sequences of relevant microsporidian taxa available in INSDC database using Geneious Pro software version 10.2.2 (https://www.geneious.com). Unrooted phylogenetic tree was inferred by the maximum likelihood (ML) method with the Tamura–Nei model or HKY + G model with MEGA X (Kumar *et al*., 2018). The tree robustness was tested by bootstrapping with 1000 replicates.

### Electron microscopy

For TEM, small infected fragments of skeletal muscle obtained from the abdomen of *P. paucidens* and fillet of *O. mykiss* were fixed in 2% glutaraldehyde and 2% paraformaldehyde in 30 mm 4-(2-hydroxyethyl)-1-piperazineethanesulphonic acid (HEPES) buffer (pH 7.4). Fixed tissues were rinsed 2 times in 30 mm HEPES buffer and post-fixed by 1% osmium tetroxide in 30 mm HEPES buffer for 1 h. Specimens were washed in 2 changes of 30 mm HEPES buffer and dehydrated through an ascending graded ethanol series prior to embedding in Poly/Bed^®^ 812 epoxy formulation (Polysciences Inc., Warrington, USA). Semi-thin sections (1.5 *μ*m) were stained with 0.5% toluidine blue for light microscopic observation to determine suitable target areas. Ultrathin sections (70 nm) were mounted on copper grids and contrasted by 2% uranyl acetate at room temperature for 15 min, and then washed with distilled water followed by being secondary-stained with lead stain solution (Sigma-Aldrich, St. Louis, USA) at room temperature for 3 min. Grids were observed using a JEM-1400Plus transmission electron microscope (JEOL Ltd., Tokyo, Japan) at an acceleration voltage of 100 kV. Digital images were shot with an EM-14830RUBY2 camera (JEOL Ltd., Tokyo, Japan).

Scanning electron microscopy (SEM) was also performed to observe the surface morphology of spores. Infected muscles of *P. paucidens* fixed in 70% ethanol were gently homogenized by BioMasher II (Nippi, Inc., Tokyo, Japan) and washed 3 times in 0.2 m phosphate buffer (Na_2_HPO_4_ and NaH_2_PO_4_) solution, pH 7.8 (PB), and immersed overnight in 2.5% glutaraldehyde in PB. The specimen was washed 3 times in PB and then post-fixed in 1% osmium tetroxide in PB for 1 h, and washed again 3 times with PB. It was placed in a 1.5 mL tube and dehydrated through a graded ethanol series, immersed in warm *t*-butyl-alcohol and cooled down in a refrigerator at 4°C for 2 h. The specimen was then freeze-dried by using JFD-300 (JEOL Ltd., Tokyo, Japan), mounted on stubs and sputter-coated with gold–palladium at 200 Å in an ion sputtering device JFC-1500 (JEOL Ltd., Tokyo, Japan). Observation of the spores was performed using a scanning electron microscope JSM-6100 (JEOL Ltd., Tokyo, Japan) at an accelerating voltage of 15 kV.

## Results

### Light microscopy of spores and histology

**Fig. 1.**
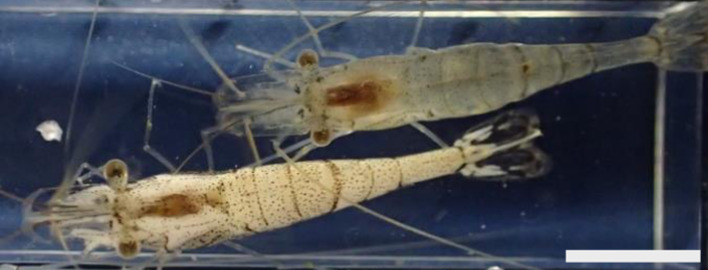
*Palaemon paucidens* showing abnormal appearance (bottom). Opacity and whitening of the skeletal muscles were obvious compared with the normal prawn (top). Scale bar: 1 cm.

**Fig. 2.**
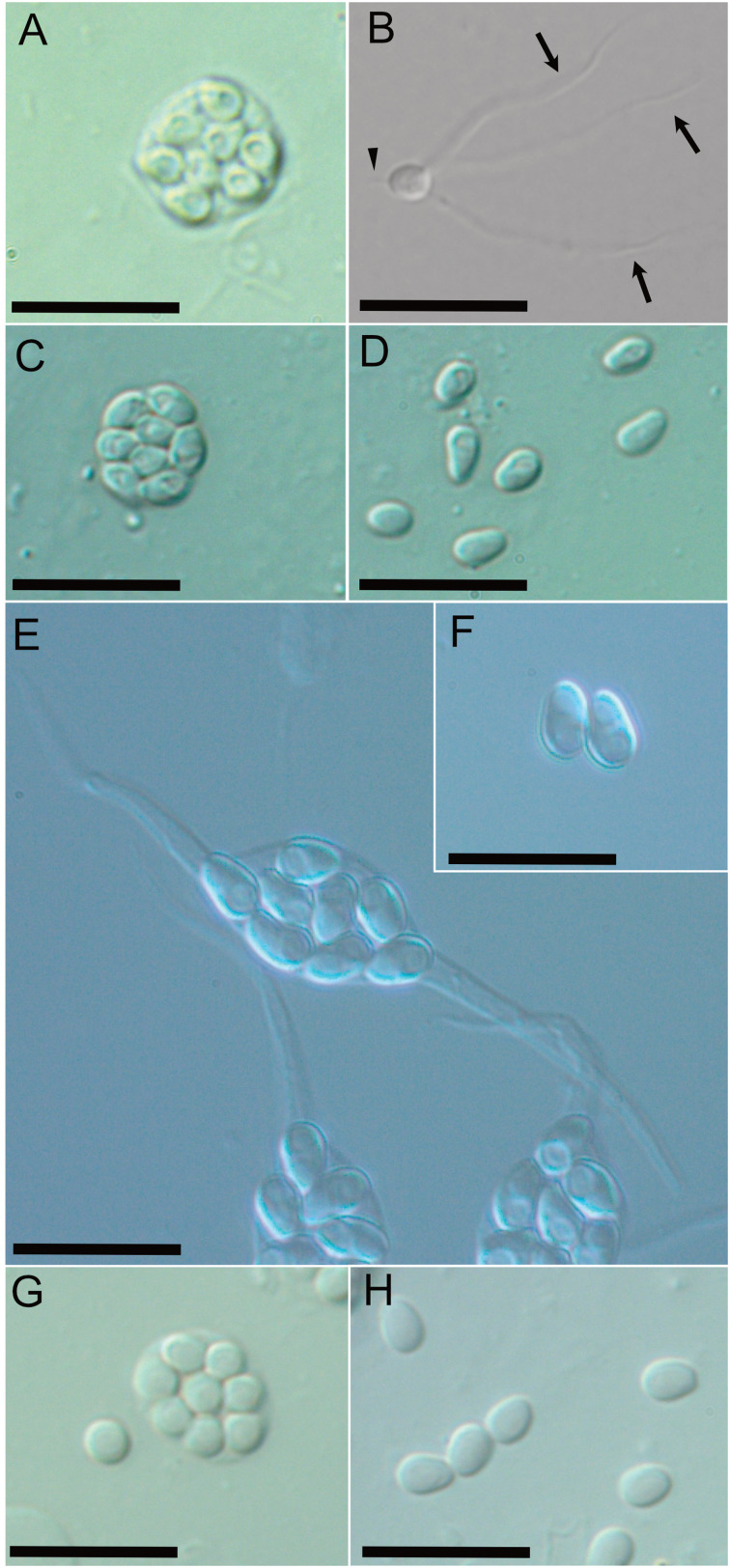
Differential interference contrast microscopy of microsporidians obtained from muscles of *P. paucidens*. Sporophorous vesicles of types 1–4 (A, C, E and G), and the spore(s) of types 1–4 (B, D, F and H). An arrowhead and black arrows (B) show anterior appendage and hair-like posterior appendages, respectively. Scale bars: 10 *μ*m.

Six out of approximately 4900 prawns collected in May 2019 were apparently opaque (Fig. 1). Examinations of these opaque *P. paucidens* revealed that all 6 were infected with microsporidian spores. Two types of microsporidian spores were confirmed from the opaque *P. paucidens* and there was a striking difference in the spore morphology (hereafter referred to as types 1 and 2). In both cases, spores were contained within a membrane in sets of mostly 8 (sporophorous vesicle), but spores of type 1 possessed 1 short and 3 long hair-like external appendages at the anterior end and the posterior sides, respectively, while type 2 has no such appendages (Fig. 2A–D). The type 1 spore was ovoid in shape (Fig. 2B), with spore length and width of 2.5 (2.0–3.0) *μ*m and 2.0 (1.7–2.5) *μ*m, respectively (Table 1). The 3 posterior external appendages were all similar in length (Fig. 2B), 20.8 (17.9–24.8) *μ*m long, representing 7.9 (6.4–9.5) times the spore length. The single anterior appendage was much shorter (Fig. 2B), almost indistinct in wet mount microscopy, and thus, no measurement was taken. The spores of type 2 were elongated oval or slightly curved (Fig. 2D) and 3.0 (2.4–3.4) *μ*m long by 1.9 (1.6–2.3) *μ*m wide (Table 1). Posterior vacuole was clearly observed in both types of microsporidian spores (Fig. 2B and D). Based on the microscopical observations, only 1 type of microsporidian was confirmed in a single *P. paucidens* (no mixed infection). No microsporidian infections were detected in the 100 individuals of non-opaque *P. paucidens* collected at the same sampling site.

**Table 1.** Comparison of microsporidian spores obtained from *Palaemon paucidens* collected in Lake Biwa in this study, and their related microsporidians

Species	*Kabatana takedai*	*Inodosporus fujiokai* n. sp.		*Inodosporus spraguei*	*Inodosporus octosporus*	*Inodosporus* sp.	*Microsporidium* sp. CP-2022A (type 2)	*Microsporidium* sp. CP-2022B (type 3)	*Microsporidium* sp. CP-2022C (type 4)
		*Microsporidium* sp. RBT-2021	Type 1						
References	Awakura (1974)	Yamamoto *et al*. (2021)	This study	Overstreet and Weidner (1974)	Azevedo *et al*. (2000)	Paschoal *et al*. (2021)	This study	This study	This study
Host	Various salmonids	*Oncorhynchus mykiss*	*P. paucidens*	*Palaemonetes pugio*	*Palaemon serratus*	*Macrobrachium amazonicum*	*P. paucidens*	*P. paucidens*	*P. paucidens*
Locality	Hokkaido, Japan; Far East Russia	Shiga, Japan	Shiga, Japan	Mississippi, USA	Atlantic coast, France and Portugal	Paraná basin, Brazil	Shiga, Japan	Shiga, Japan	Shiga, Japan
Spore length (*μ*m)	3.4 (2.8–4.9)	2.9 (2.7–3.0)	2.5 (2.0–3.0)	2.9 (2.0–3.7)	2.6 (2.4–2.8)/3.8 (3.5–4.2)	4.3 (4.1–4.7)	3.0 (2.4–3.4)	4.3 (3.6–4.8)	3.3 (2.9–3.8)
Spore width (*μ*m)	2.0 (1.7–2.3)	1.9 (1.7–2.0)	2.0 (1.5–2.6)	2.0 (1.7–2.5)	1.3 (1.2–1.5)/2.4 (2.0–2.9)	3.7 (3.3–4.1)	1.9 (1.6–2.3)	2.5 (2.1–2.9)	2.2 (1.9–2.5)
Length/width	NA	1.5 (1.4–1.7)	1.3 (1.1–1.5)	NA	NA	NA	1.6 (1.3–2.0)	1.4 (1.3–2.2)	1.6 (1.3–1.8)
Filament coils	3–4^a^	4–5	5–6	4–5	5–6	7–8	NA	NA	NA
Spore shape	Ovoid to elongated oval	Ovoid to elongated oval	Ovoid	Pyriform	Ellipsoidal	Pyriform to ovoid	Elongated oval	Ovoid to elongated oval	Ovoid to ellipsoidal
External appendages	Absent	Absent	Present	Present	Present	Present	Absent	Absent	Absent
Sporophorous vesicle	Absent	Absent	Spherical	Spherical	Spherical	Spherical	Spherical	Elongated spindle	Spherical

NA, not available.

a The data cited from Lom *et al*. (2001).

In June 2020, a total of 26 opaque *P. paucidens* was collected and examined for microsporidian infection. Microsporidian spores were detected in all of those prawns, and in addition to types 1 and 2, 2 more microsporidians which were different in wet mount light microscopy were confirmed (types 3 and 4). Spores of type 3 were also contained within sporophorous vesicles in sets of 8. In comparison with the spherical sporophorous vesicle of types 1, 2 and 4, that of type 3 was clearly distinguishable by its elongated spindle shape (Fig. 2E). The spores of type 3 were ovoid to elongated oval and often slightly curved (Fig. 2F), with spore length and width of 4.3 (3.6–4.8) *μ*m and 2.5 (2.1–2.9) *μ*m, respectively (Table 1). Posterior vacuole was clearly visible. Type 4 spores in spore vesicles, in a set of mostly 8, were ovoid to ellipsoidal and morphologically resembled to those of type 2, with slightly larger but overlapping size range of 3.3 (2.9–3.8) *μ*m long by 2.2 (1.9–2.5) *μ*m wide (Table 1). Among the 26 infected *P. paucidens*, type 1 was predominant (65.4%), followed by type 4 (15.4%), type 2 (11.5%) and type 3 (7.7%).

**Fig. 3.**
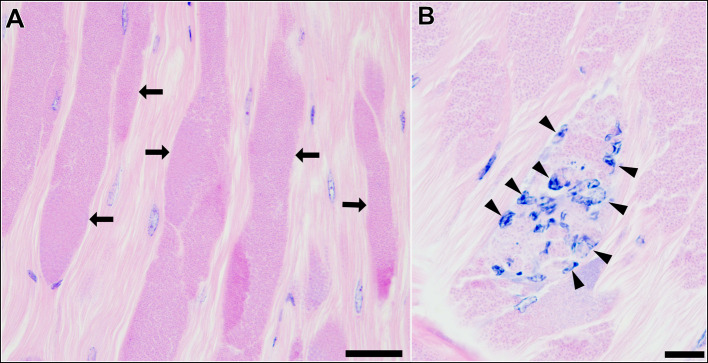
Longitudinal sections of the skeletal muscle of *P. paucidens* infected with *Inodosporus fujiokai* n. sp. (type 1). H&E stains. (A) Masses of microsporidian spores and other developmental stages (arrows) replace normal muscle fibres. (B) Infiltration of host haemocytes (arrowheads). Scale bars: 50 *μ*m for (A); 20 *μ*m for (B).

Histological observation of the skeletal muscle tissue infected with type 1 revealed that a large part of muscle fibres and myofibrils were replaced by masses of microsporidian spores and other developmental stages (Fig. 3A). Infiltration of host haemocytes was only occasionally observed (Fig. 3B).

### Molecular analysis

From type 1 microsporidians in *P. paucidens*, 1333 bp of SSU rDNA sequence was obtained. It was 100% identical (1333/1333) to that of *Microsporidium* sp. RBT-2021 (LC612731) from *O. mykiss*. BLAST search revealed that the rDNA of *K. takedai* (AF356222) infecting masu salmon *Oncorhynchus masou* from Hokkaido, Japan was the second closest, with an identity of 98.2%. For type 2, type 3 and type 4 microsporidians from *P. paucidens*, 1807, 1705 and 1664 bp of rDNA sequences were determined, respectively. The nucleotide sequence of type 2 was closest to that of *Potaspora macrobrachium* (KU307278), a microsporidian recently described from the freshwater prawn *Macrobrachium nipponense* in China (Ding *et al*., 2016) with an identity of 99.3% (821/827). Type 3 was closest to *Nosema apis* (FJ789794), the pathogen of nosemosis in honey bees *Apis mellifera*, with an identity of 95.4% (658/690, with 1 gap). Type 4 was closest to *Potaspora* sp. MMM-2017a (MF681638), which was found in a palaemonid shrimp *Palaemonetes argentinus* in Argentina with an identity of 98.6% (726/736). Among the 4 newly found microsporidians from *P. paucidens* in Lake Biwa in this study, similarity in the nucleotide sequences of types 2 and 4, with similar spore morphology, was relatively high (83.9%). On the other hand, type 3 was distantly related to other 3 microsporidians, with the pairwise similarity of only 54.8–56.2%. For *M. seriolae*, 1328 bp of SSU rDNA sequence was determined. It was 99.4% similar (890/895, with 5 gaps) to *M. seriolae* from the yellowtail *Seriola quinqueradiata* in Japan (AJ295322). The nucleotide sequences obtained in this study are available in INSDC database under the accession numbers LC704895–LC704899.

**Fig. 4.**
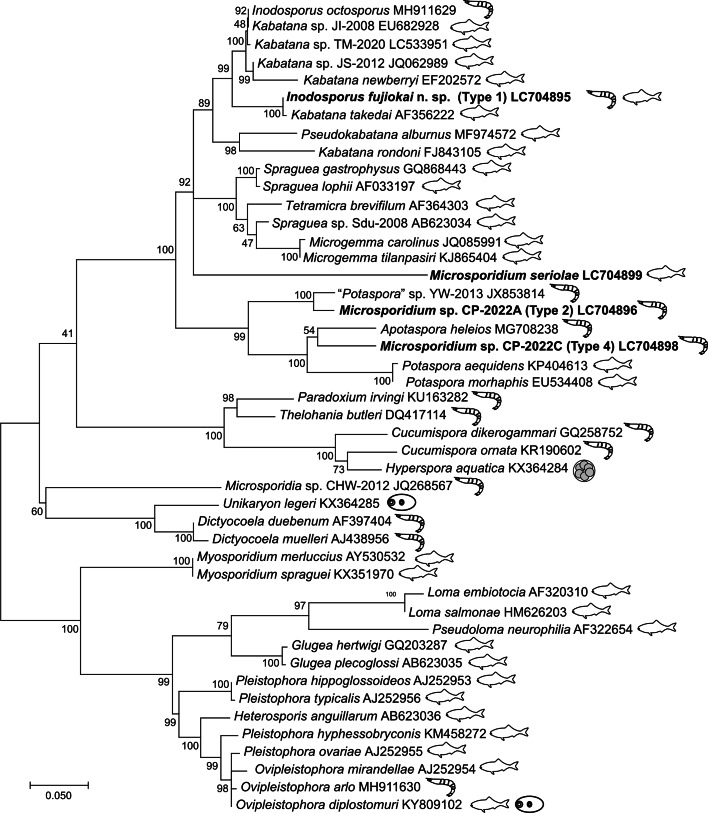
Phylogenetic tree inferred by the ML method based on the alignment of 826 nucleotide positions in SSU rDNA. The taxa shown in bold are the sequences determined in this study. Numbers on the nodes are the bootstrap value. Hosts of each microsporidian species are shown as symbols (fish, crustacean, digenean and paramyxida).

**Fig. 5.**
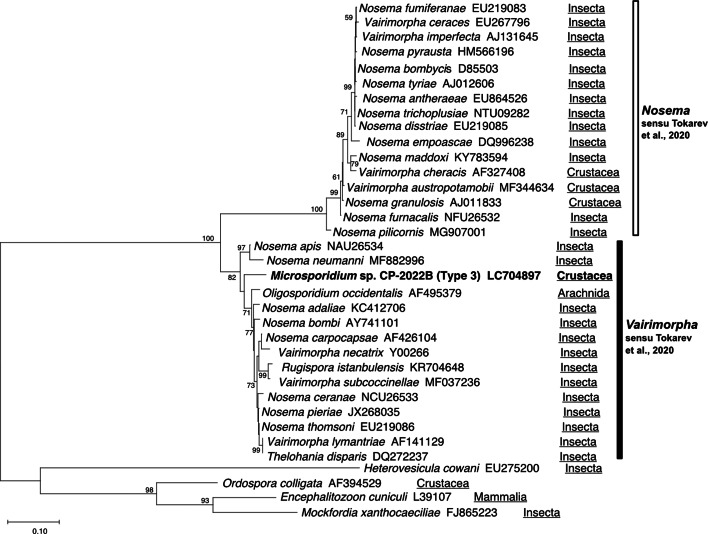
Phylogenetic tree inferred by the ML method based on the alignment of 1034 nucleotide positions in SSU rDNA. The taxon shown in bold is the sequence determined in this study. Numbers on the nodes are the bootstrap value (only >50 are shown). Hosts of each microsporidian species are shown underlined.

In the preliminary phylogenetic analysis, it was shown that types 1, 2 and 4 were placed within the ‘clade 5 (Marinosporidia)’ of the main 5 clades in Microsporidia, while type 3 was placed within ‘clade 4 (Terresporidia)’ (Vossbrinck *et al*., 2014). Therefore, we performed 2 independent phylogenetic analyses instead of conducting a large-scale phylogenetic analysis including all 4 types. It was demonstrated that types 1, 2 and 4 were placed within the clade comprising microsporidians of fish and crustaceans (Fig. 4). Type 1 clustered with *Kabatana* spp. and *I. octosporus* with high bootstrap value (99%). *Kabatana rondoni* and *Pseudokabatana alburnus* made a sister clade to the one with type 1, but the bootstrap value of the node was <95% (89%). Types 2 and 4 were placed in the robust clade consisting of *Potaspora* spp. and *Apotaspora heleios* with high bootstrap value (99%). Although *P. macrobrachium* (KU307278) and *Potaspora* sp. MMM-2017a (MF681638) were closest to types 2 and 4, respectively, in the BLAST search, they were excluded from the analysis because their overlapping regions with other taxa were considerably short. *Microsporidium seriolae* was sister to both the clades containing *Kabatana* spp. and the one comprising *Spraguea* spp., *Microgemma* spp. and *Tetramicra brevifilum*. Type 3 was found to be related to the microsporidians of insects or arachnids, such as *Nosema* spp. and *Vairimorpha* spp., and placed within the clade recently defined as the genus *Vairimorpha* by Tokarev *et al*. (2020) (Fig. 5).

### Electron microscopy

**Fig. 6.**
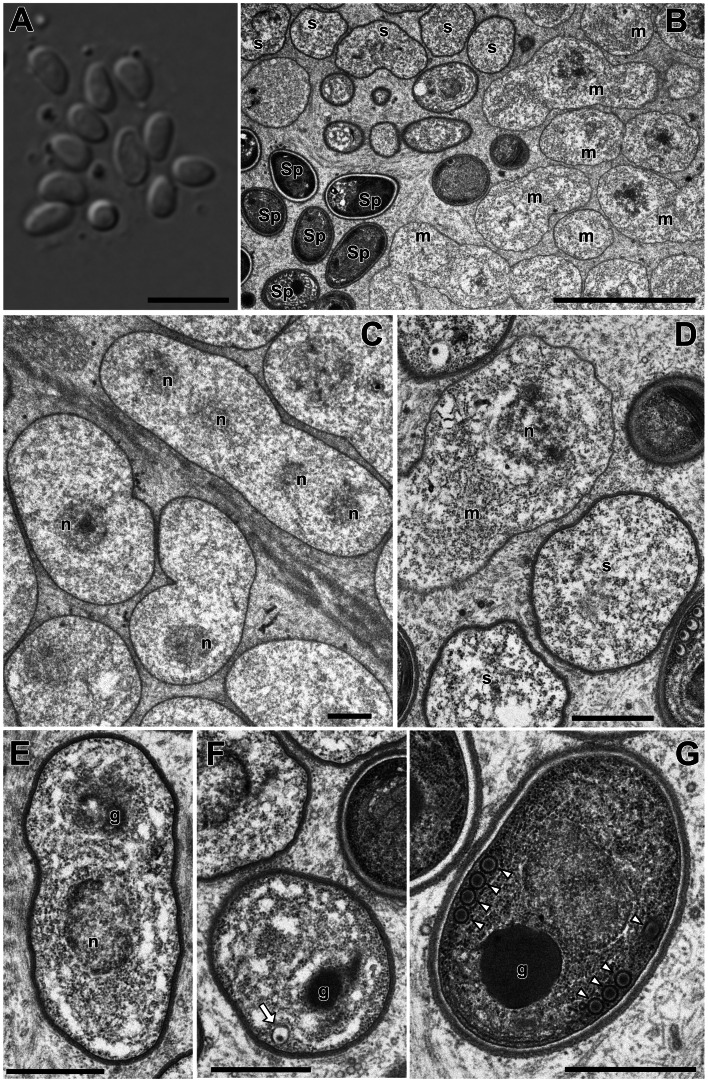
Light microscopy (A) and electron microscopy (B–G) of *I. fujiokai* n. sp. in the somatic muscle of *Oncorhynchus mykiss*. (A) Fresh spores. (B) Developmental stages. Meronts (m), sporonts (s) and immature and mature spores (Sp) are simultaneously observed in the host cell. (C) Uninucleate meronts and a cylindrical multinucleate meronts. n, parasite nuclei. (D) Sporonts with the plasmalemma apparently thicker than that of meronts. (E, F) Early sporoblast with a dense globule (g). Arrow indicates a primordium of the polar filament. (G) Immature spore with a dense globular inclusion (g) and the cross section of 4 coils of the polar filament (arrowheads). Scale bars: 5 *μ*m for (A) and (B); 1 *μ*m for (C–G).

**Fig. 7.**
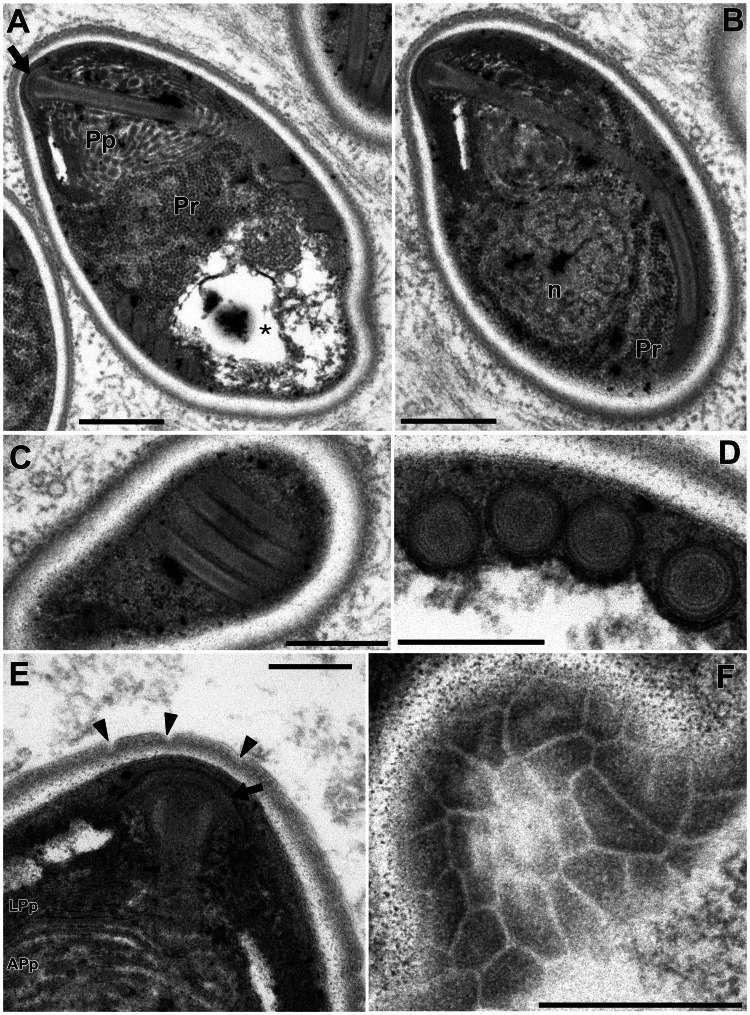
Electron microscopy of *I. fujiokai* n. sp. in the somatic muscle of *O. mykiss*. (A) Mature spore with subterminal position of the anchoring disc (arrow). The polaroplast (Pp) situated posterior to the anchoring disc, and the mass of polyribosomes (Pr) was observed anterior to the posterior vacuole (asterisk). (B) Mature spore with obliquely extending polar filament. A round nucleus (n) in the central position surrounded by the strands of polyribosomes (Pr). (C) Mature spore showing 4 coils of the polar filament. (D) Transverse section of 4 coils of the polar filament. (E) Anchoring disc (arrow) of the spore and the lamellar and alveolar polaroplast (LPp and APp). Arrowheads indicate the furrows dividing the exospore into small fields. (F) Grazing section of the exospore. Scale bars: 500 nm for (A–C, F); 200 nm for (D) and (E).

In this study, only *Microsporidium* sp. RBT-2021 and type 1 were examined by TEM. In the infected trunk muscle of *O. mykiss*, various developmental stages including spores of *Microsporidium* sp. RBT-2021 were observed within a host cell (Fig. 6B). In the fish host, sporophorous vesicle was absent during all life cycle stages. The earliest stage was a uninucleate meront, subsequently developing to a long multinucleate cylinder shape with serially arranged nuclei (Fig. 6C). The multinucleate meront divided by plasmotomy or binary fission into uninucleate cells. Those cells transformed into sporonts with the plasmalemma apparently thicker than that of meronts (Fig. 6D). The sporont seemed to develop directly into sporoblast, as the cell division of the sporont was not observed. In the early sporoblasts, a dense globule was observed which persisted until the immature spore (Fig. 6E–G). The primordium of the polar filament also appeared in the early sporoblast (Fig. 6F). In the mature spore, the anchoring disc was subterminal and the isofilar polar filament extended obliquely backwards to the spore wall (Fig. 7A and B) and coiled 4 (rarely 5) times in 1 row (Fig. 7C and D). The polaroplast situated posterior to the anchoring disc, and reached to about 1/3 of the spore length. The anterior part of the polaroplast was a stack of thin lamellae, and the posterior part consisted of flat alveoli (Fig. 7A and E). The round nucleus with irregularly dispersed aggregates of chromatin occupied a central position in the spore (Fig. 7B). The sporoplasm contained masses or strands of polyribosomes surrounding the nucleus (Fig. 7A and B). The surface of the exospores was divided into small irregular fields (Fig. 7E and F).

**Fig. 8.**
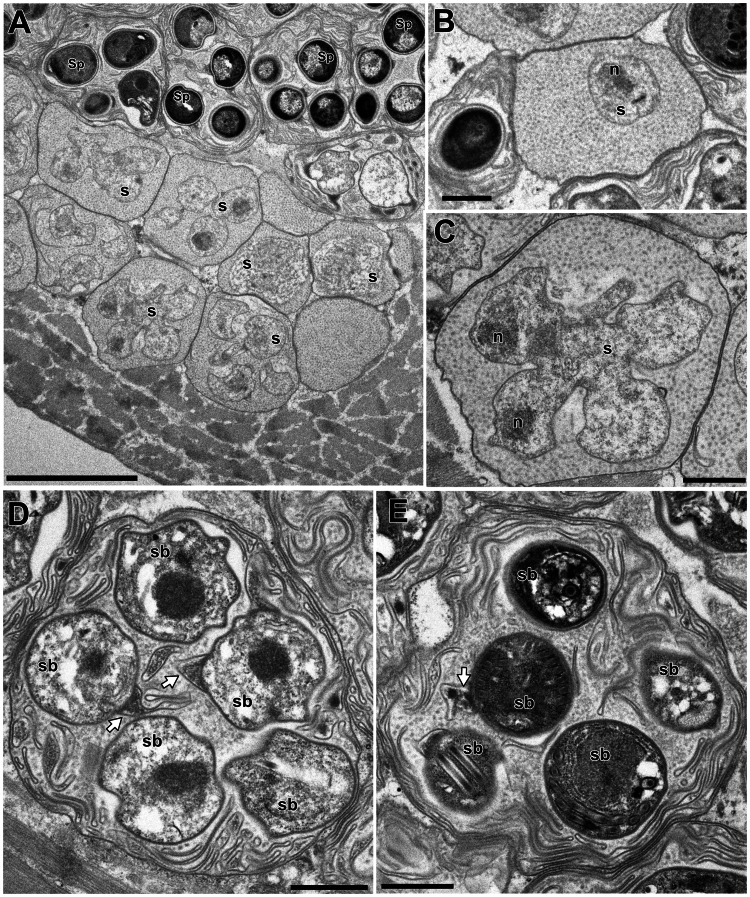
Electron microscopy of *I. fujiokai* in the muscle of *P. paucidens*. (A) Sporonts (s) and spores (Sp) simultaneously observed in the muscle myofibre. (B) Uninucleate sporont within sporophorous vesicle. n, nucleus. (C) Sporophorous vesicle containing a rosette sporont with nuclei. (D, E) Sporophorous vesicle containing sporoblasts showing tape-like filaments in direct contact with sporoblast walls (arrows). Scale bars: 5 *μ*m for (A); 1 *μ*m for (B–E).

**Fig. 9.**
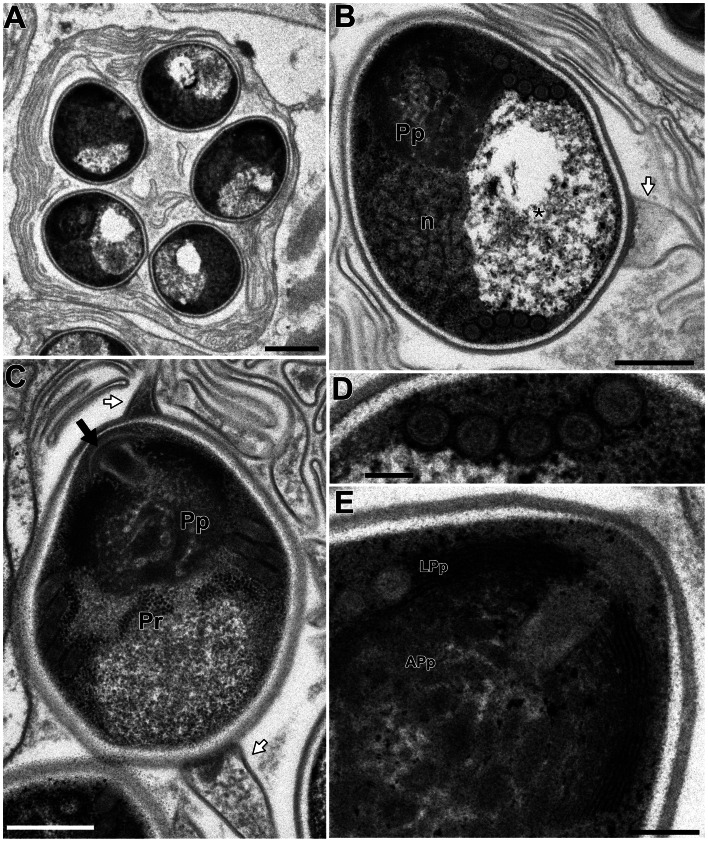
Electron microscopy of *I. fujiokai* in the muscle of *P. paucidens*. (A) Sporophorous vesicle containing mature spores. (B) Mature spore with polaroplast (Pp), nucleus (n) and posterior vacuole (asterisk). White arrow indicates the proximal regions of the tape-like external appendage. (C) Mature spore with subterminal position of the anchoring disc (black arrow). The polaroplast situated posterior to the anchoring disc, and the strands of polyribosomes (Pr) are observed. (D) Transverse section of 5 coils of the polar filament. (E) Anchoring disc of the spore and the anterior lamellar and the posterior alveolar polaroplast (LPp and APp). Scale bars: 1 *μ*m for (A); 500 nm for (B) and (C); 100 nm for (D); 200 nm for (E).

**Fig. 10.**
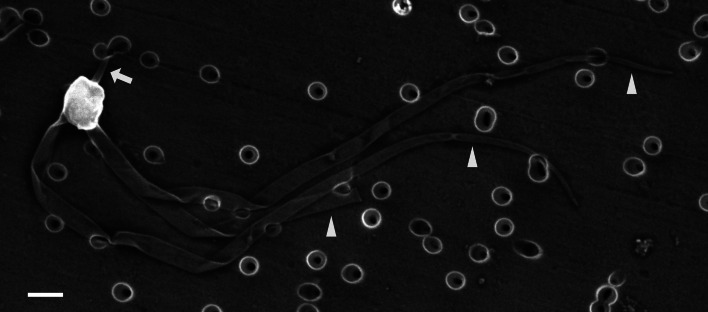
SEM of the spore of *I. fujiokai* n. sp. with a short anterior appendage (arrow) and 3 posterior appendages (arrowheads), collected from the abdominal muscle of *P. paucidens*. Note one of the posterior appendages is torn off. Scale bar: 1 *μ*m.

In the infected muscle of *P. paucidens*, the sporonts and spores of type 1 were simultaneously observed within myofibres (Fig. 8A). All those stages were found within a sporophorous vesicle, separated from the organelles and cytoplasm of the host cell. In this study, we could not confirm meronts, which were shown to be uninuclear or diplokaryotic cells directly in contact with the host cells in *Inodosporus* spp. (Overstreet and Weidner, 1974; Azevedo *et al*., 2000). We also could not observe a diplokaryon state of the early sporont, and the earliest sporogonic stage was a uninucleate cell within the sporophorous vesicle (Fig. 8B). Sporogony proceeded by budding and finally produced commonly 8 uninucleate sporoblasts, although the maximum number of sporoblasts observed in each ultrathin section was 5 (Fig. 8C and D). Fine-granular substances filled the space between the sporont wall and the interfacial membrane of the sporophorous vesicle. During the development of sporoblasts, membranous elements appeared within the sporophorous vesicle and some of them were observed in close contact with sporoblasts (Fig. 8D and E). The membranous elements were evident through the development of mature spores (Fig. 9A), and protrusion of the external appendages on the surface of spores were confirmed (Fig. 9B and C). In mature spores, the anchoring disc was subterminal and the isofilar polar filament coiled 5 (rarely 6) times (Fig. 9B–D). The polaroplast almost reached half the spore length, and it consisted of anterior lamellar and posterior alveolar parts (Fig. 9E). The nucleus was situated at the centre of the spore, and the masses or strands of polyribosomes were observed in the sporoplasm (Fig. 9B and C). In SEM observation, the external appendages of type 1 spores were tape-like and the posterior ones gradually tapered towards pointed tips, while the anterior short one ended flat (Fig. 10).

## Discussion

In the present study, a multi-host life cycle utilizing fish and crustacean hosts was demonstrated for a microsporidian *Microsporidium* sp. RBT-2021, which has recently been described as the cause of a novel microsporidian disease of *O. mykiss* farmed in Japan. As the diseased fish have a history of being fed with prawns, mostly *P. paucidens*, caught in Lake Biwa, its involvement in the transmission of the disease was suspected. Experimental infection by feeding fresh *P. paucidens* successfully reproduced the disease in *O. mykiss*, although the microsporidian infection in the prawns was not confirmed (Yamamoto *et al*., 2021). In this study, 4 different microsporidians were found in the musculature of *P. paucidens* collected in Lake Biwa. This is the first report of microsporidian infection in *P. paucidens*. Molecular analysis revealed that 1 of them (type 1) has an identical SSU rDNA sequence to *Microsporidium* sp. RBT-2021. Together with the results of experimental infections reported by Yamamoto *et al*. (2021), we conclude that *Microsporidium* sp. RBT-2021 and type 1 are conspecific and both fish and crustacean hosts are involved in the life cycle of this microsporidian species. Intriguingly, however, the morphology of the spores was strikingly different in fish and crustacean hosts. While the spores of type 1 possess the external appendages and develop within the sporophorous vesicles (Fig. 2A and B), those of *Microsporidium* sp. RBT-2021 lack appendages and sporophorous vesicles (Fig. 6A). Such dimorphic or polymorphic development producing morphologically distinct spores in the different hosts has been demonstrated for microsporidians infecting mosquitos (Becnel *et al*., 2005; Andreadis *et al*., 2013, 2018). Among microsporidians infecting fish, *Desmozoon lepeophtherii* has been isolated both from a parasitic copepod *Lepeophtheirus salmonis* and its host Atlantic salmon *Salmo salar*, and the spore morphology and mode of development are different in each host (Nylund *et al*., 2010; Freeman and Sommerville, 2011). Although the transmission between the copepod and fish hosts has not been experimentally confirmed, polymorphic 2-host life cycle has been suggested for *D. lepeophtherii*. In the case of *Microsporidium* sp. RBT-2021, results of this study and the previous prawn-feeding trials strongly suggest the species has a multi-host life cycle. This finding gives us an important insight into the life cycle of some important fish microsporidians hidden under a veil of mystery, such as in the cases of *K. takedai* and *M. seriolae*.

While the experimental infections from the prawn to fish were successful (Yamamoto *et al*., 2021), the transmission from fish to prawn has not been demonstrated yet. It is necessary to conduct infection experiments from fish to prawn, as well as fish to fish and prawn to prawn, to elucidate the life cycle of *Microsporidium* sp. RBT-2021. To date, *Microsporidium* sp. RBT-2021 has only been confirmed in the cultured or experimentally infected *O. mykiss* and Biwa trout *O. masou* subsp. (*O. masou rhodurus*), an endemic subspecies in Lake Biwa, but never been reported from wild-caught fish. As *O. mykiss* is not indigenous to Japan and rarely occurred, if any, in Lake Biwa, there must be fish host(s) other than *O. mykiss* maintaining the life cycle of *Microsporidium* sp. RBT-2021 in the lake. *Oncorhynchus masou* subsp. is the most abundant salmonid fish in the lake and thus the most probable candidate. Further investigation is needed to clarify the natural fish host(s) in Lake Biwa.

The SSU rDNA nucleotide sequence of *Microsporidium* sp. RBT-2021 (=type 1) showed 98.2% similarity to that of *K. takedai*, and the molecular phylogenetic analysis clearly indicated that the species has a genetic affinity to *Kabatana* spp., a group of microsporidians infecting the muscle of fish. In the electron microscopic observation, *Microsporidium* sp. RBT-2021 in *O. mykiss* showed the characteristics of the genus *Kabatana*, e.g. nuclei are isolated at all the developmental stages, development in direct contact with host cell cytoplasm (lack of sporophorous vesicle), cylindrical multinucleate meronts dividing by plasmotomy and binary fission and spore surface is divided into small fields (Lom *et al*., 1999). Besides, the development of *Microsporidium* sp. RBT-2021 in the host cell was mostly similar to that of *K. takedai*, except for the lack of cell division of the sporonts before becoming sporoblasts and the number of coils of the polar filament (Lom *et al*., 2001). Thus, molecular and ultrastructural analyses in this study undoubtedly indicate *Microsporidium* sp. RBT-2021 belongs to the genus *Kabatana*. Among the known *Kabatana* species, *K. takedai* is the only species reported in Japan, and its host fish species (salmonids) overlaps with *Microsporidium* sp. RBT-2021. However, reported geographical distribution of *K. takedai* is restricted to certain water systems in Hokkaido, the northern island of Japan and Sakhalin, Russia (Urawa and Awakura, 1994; Vyalova, 1999), and has never been confirmed in other areas. Moreover, the genetic difference between *Microsporidium* sp. RBT-2021 and *K. takedai* (1.8% in SSU rDNA) is greater than those found among some fish microsporidians such as *Kabatana newberryi vs Kabatana* sp. JI-2008 (1.5%) and among *Spraguea* spp. (0.3–0.9%) (Barber *et al*., 2009; Casal *et al*., 2012). These facts indicate *Microsporidium* sp. RBT-2021 is an undescribed species in the genus *Kabatana*. On the other hand, the spores of type 1 (=*Microsporidium* sp. RBT-2021) from *P. paucidens* develop in group of 8 within sporophorous vesicles and have the tape-like external appendages, which are the typical characteristics of the genus *Inodosporus* (Overstreet and Weidner, 1974; Azevedo *et al*., 2000; Paschoal *et al*., 2021), the microsporidians of crustacea. Our electron microscopic observation also supported that type 1 from *P. paucidens* is a member within the genus, although we could not confirm the merogony in this study. To date, only 2 valid and 1 unspecified species have been described in the genus *Inodosporus* and none of them has been reported in Japan. The nucleotide sequence of SSU rDNA is only available for *I. octosporus*, and its similarity to that of type 1 is 84.5%. The spore morphometrics of *Inodosporus spraguei* resembles to type 1, but they are distinguishable as the anterior external appendage of the former species branches to form 2 or more filaments (Overstreet and Weidner, 1974), while that of the latter does not branch. *Inodosporus* sp. from Amazonian River prawn *Macrobrachium amazonicum* is morphologically distinguishable by its larger spores and greater number of filament coils. Thus, we conclude that *Microsporidium* sp. RBT-2021 is a novel species possessing the characteristics of both the genera *Kabatana* and *Inodosporus*, in *O. mykiss* and *P. paucidens*, respectively. The genus *Kabatana* was originally erected as *Kabataia* in 1999 by Lom *et al*. (1999) and later replaced in 2000 (Lom *et al*., 2000), while the genus *Inodosporus* was erected in 1974 by Overstreet and Weidner (1974). Therefore, we describe *Microsporidium* sp. RBT-2021 as *Inodosporus fujiokai* n. sp., placing in the older genus.

This study strongly suggests that the genus *Kabatana* is a junior synonym of the genus *Inodosporus* by the principle of priority. Recently, Stentiford *et al*. (2018) also suggested their synonymy based on the SSU rDNA sequence similarity between *Kabatana* sp. JI-2008 and *I. octosporus*. The result of molecular phylogeny in this study also supports the idea that these genera should be unified. However, the nucleotide sequences of *Kabatana arthuri* and *I. spraguei*, the type species of the genera *Kabatana* and *Inodosporus*, respectively, have not been determined until now. Phylogenetic analysis including these taxa would provide us with an important clue to make a conclusion on the taxonomic status of those genera. Whether those genera would be synonymized or not, the results of this study led us to presume that the microsporidians belonging to the genera *Inodosporus*/*Kabatana* have dimorphic or polymorphic life cycles utilizing multiple hosts, i.e. fish and crustacean hosts. Fujiyama *et al*. (2002) reported that the experimental challenges of *K. takedai* to *O. mykiss* using spores collected from the infected fish were not successful, while masu salmon *O. masou* exposed to infectious river water filtered by nylon meshes with openings of 40 *μ*m were infected. Based on these results, it was suggested that the transmission of *K. takedai* to fish is due to the unknown infectious stages smaller than 40 *μ*m. It is speculated that the spores of *K. takedai* released from alternate host(s) could pass through this mesh size, even if they have external appendages like as the other *Inodosporus* spp. It would be intriguing to conduct a parasitological investigation targeting crustaceans, especially freshwater prawns, in the endemic areas of *K. takedai*.

Histological observation revealed that the opaque skeletal muscle of *P. paucidens* infected with *I. fujiokai* n. sp. was filled with masses of spores and other developmental stages. Regardless of the fact that the almost entire muscle tissue was affected, the opaque *P. paucidens* were apparently healthy and some even harboured eggs. These opaque *P. paucidens* survived under captivity for several weeks and thus, *I. fujiokai* n. sp. seems not to be highly pathogenic to the crustacean host. On the other hand, high mortality was observed in *O. mykiss* infected with *I. fujiokai* n. sp. (Yamamoto *et al*., 2021).

In this study, a total of 4 microsporidians were confirmed in the musculature of *P. paucidens* from Lake Biwa. Types 2 and 4 are morphologically similar, but there is a 16.1% difference in the rDNA, indicating they are distinct species. In the ML tree, these 2 species were placed within the robust clade comprising *Potaspora* and *Apotaspora* species. The species included in this clade have a fish or a crustacean host, and the taxonomic status of some species need verification. The genus *Potaspora* was erected for a novel xenoma-forming microsporidian *Potaspora morhaphis* found from an Amazonian fish in Brazil (Casal *et al*., 2008), and the second species *Potaspora aequidens* was also described for a xenoma-forming microsporidian infecting another Amazonian fish (Videira *et al*., 2015). Ding *et al*. (2016) described the third species *P. macrobrachium* which was found from a freshwater prawn in China, although it does not form a xenoma. A question was later raised whether *P. macrobrachium* should be included in the genus or not. Sokolova and Overstreet (2018) pointed that *P. macrobrachium* was placed in the genus based exclusively on the genetic affinity, even though the sequence identity of 87% between the SSU rDNA of *P. macrobrachium* and *P. morhaphis* does not necessarily mean they belong to the same genus. They erected a new genus *Apotaspora* for a microsporidian *A. heleios* from the Riverine grass shrimp *Palaemonetes paludosus*, and stated that ‘the confusion caused by a polyphyletic taxon will be corrected by the creation of a new combination for *P. macrobrachium* in future revisions of the genus *Potaspora* and related taxa’ (Sokolova and Overstreet, 2018, p. 133). Ultrastructural analyses on types 2 and 4 in future would help us to resolve the confusion on the taxonomy of *Potaspora*-related taxa. Until then, we tentatively designate type 2 and type 4 as *Microsporidium* sp. CP (common prawn)-2022A and *Microsporidium* sp. CP-2022C, respectively.

Among the 4 microsporidians confirmed from *P. paucidens*, type 3 is unique in terms of its morphology and phylogenetic position. The sporophorous vesicle of type 3 is elongated spindle-shaped, which makes this species easily distinguishable from the other 3 microsporidians. Sporophorous vesicles of known microsporidian species are normally spherical or ovoid with a few exceptions. Microsporidians belonging to the genera *Chapmanium*, *Napamichum* and *Ormieresia* develop spores in fusiform sporophorous vesicles (Vávra and Larsson, 2014). The known species in those genera have mostly been reported from insects and none of them possesses such elongated spindle-shaped sporophorous vesicles (Lipa, 1966; Hazard and Oldacre, 1975; Vivarès *et al*., 1977; Larsson, 1984, 1990; Bylén and Larsson, 1994), except for *Chapmanium macrocystis*. This species was first reported by Garbini (1891) as a novel sarcosporidian parasite infecting the muscle of *Palaemon varians* in Italy, and later described as *Thelohania macrocystis* by Gurley (1893, 1894) using the data from Garbini (1891). Hazard and Oldacre (1975) replaced this species within the genus *Chapmanium* because of its fusiform shape of sporophorous vesicle containing 8 spores, but only tentatively, as the original description was incomplete. The drawings of the sporophorous vesicle in Garbini (1891) closely resemble to type 3, but unfortunately, the morphometric data of *C. macrocystis* has not been provided until now. Our phylogenetic analysis showed that type 3 is genetically related to the microsporidians infecting insects, such as *Nosema* spp. and *Vairimorpha* spp. Recently, the polymorphic genera *Nosema* and *Vairimorpha* were redefined based on the molecular phylogenetics using SSU rDNA sequences (Tokarev *et al*., 2020). The genus *Nosema* was defined as a group of microsporidians showing ⩾94% similarity to the SSU rDNA of the type species *Nosema bombycis* (D85503), while the genus *Vairimorpha* as ⩾91% to *Vairimorpha necatrix* (U11051) (Tokarev *et al*., 2020). According to the definition, type 3 can be placed in the genus *Vairimorpha*, as the sequence similarity to *V. necatrix* was 92.8%. In the ML tree, type 3 was also placed within the clade of the genus *Vairimorpha* sensu (Tokarev *et al*., 2020). However, its sister taxon in the ML tree and the mode of development is unknown for type 3, and thus, we herein tentatively designate it as *Microsporidium* sp. CP-2022B. These results indicate the unexpectedly high diversity of microsporidians in *P. paucidens* in Lake Biwa. Although *P. paucidens* are commercially fished for local cuisine around the lake, microsporidian infection has been overlooked to date. Lake Biwa is the largest in Japan, and one of the ancient lakes of the world, which has existed for millions of years (Cristescu *et al*., 2010). It harbours rich fauna and flora including more than 60 endemic species/subspecies (Tabata *et al*., 2016), and thus further investigations on *P. paucidens* and other crustaceans in Lake Biwa may provide more information on microsporidian biodiversity. Our previous prawn-feeding experiments to *O. mykiss* and *O. masou* subsp. resulted in the infections of only *I. fujiokai* n. sp. (type 1). Other microsporidians found in *P. paucidens* (types 2–4) may not be infective to these fishes. The elucidation of their life cycles, including the utilization of multiple or single host(s), will provide further insight into the biology of microsporidians.

In this study, the SSU rDNA sequence of *M. seriolae* was also determined, and its phylogenetic position was evaluated. Egusa (1982) described *M. seriolae* as the cause of ‘beko’ disease of *S. quinqueradiata*, but not identified at the species level because it did not fit into any known genus at that time. Later, Lom *et al*. (1999) proposed to reassign the species into the genus *Kabatana* based on the mode of development, although the merogony stages were not described in the original description (Egusa, 1982). It has thereafter been referred to as ‘*Kabatana seriolae*’ in some cases (McGourty *et al*., 2007; Barber *et al*., 2009; Casal *et al*., 2010; Liu *et al*., 2019). However, it has often been shown that *M. seriolae* and *Kabatana* species does not make a monophyletic clade (McGourty *et al*., 2007; Casal *et al*., 2010; Liu *et al*., 2019). The available SSU rDNA sequences of *M. seriolae* in the INSDC database lack the first 300–400 nucleotides in the 5′ end (Bell *et al*., 2001; Mekata *et al*., 2021) and this may decrease phylogenetic resolution by shortening the resultant alignment dataset. In this study, we succeeded to determine the lacking region and obtained almost full length of the SSU rDNA (1328 bp) for *M. seriolae*. With this newly determined sequence in the ML tree, *M. seriolae* did not make a monophyletic clade with *Kabatana* spp. and polytomy among *M. seriolae*, *Inodosporus*/*Kabatana*/*Pseudokabatana* lineage and *Microgemma*/*Spraguea*/*Tetramicra* lineage was found. From the phylogenetic perspective based on the SSU rDNA sequences, *M. seriolae* should not be placed into the genus *Kabatana* at the moment. To clarify the taxonomic position of *M. seriolae*, further phylogenetic analyses including microsporidians related to *M. seriolae*, presumably those infecting muscle of marine fish, would be essential.

## Taxonomic summary

*Inodosporus fujiokai* n. sp. Yanagida, Asai, Yamamoto, Sugahara, Fujiwara, Shirakashi and Yokoyama

*Phylum*: Microsporidia

*Family*: Thelohaniidae Hazard and Oldacre, 1975

*Genus*: *Inodosporus* Overstreet and Weidner, 1974

*Description*: Infects myofibres of *P. paucidens* and *O. mykiss* and *O. masou* susp. Spore morphology and mode of development are different in *P. paucidens* and *O. mykiss*. In *P. paucidens*, monokaryotic ovoid spores (2.5 × 2.0 *μ*m) with 1 short anterior and 3 long posterior tape-like external appendages (average 20.8 *μ*m). Six to 8 spores (8 spores: 56%) within sporophorous vesicle (5.6 × 6.8 *μ*m). Polar filament coiled 5–6 turns arranged in 1 row. Merogony not confirmed. Sporogony proceeds by budding and produces uninucleate sporoblasts within sporophorous vesicle. Sporophorous vesicles are filled with fine-granular substances during the early sporogony, then membranous elements appear and are evident through the development of mature spores. In *O. mykiss*, sporophorous vesicle is absent during all life cycle stages. Monokaryotic ovoid to elongated oval spores (2.9 × 1.9 *μ*m) having a polar filament with 4–5 coils in 1 row. The exospore surface is divided into small irregular fields. Merogony involves uninucleate and multinucleate cylinder-shaped meronts. Multinucleate meronts divided into uninucleate cells, subsequently transformed into sporonts with thicker plasmalemma. Sporonts directly develop into sporoblasts having a dense globule which persists until the immature spores.

*Type host*: Common prawn *Palaemon paucidens*

*Other hosts*: Rainbow trout *Oncorhynchus mykiss* (experimental), Biwa trout *Oncorhynchus masou* subsp. (experimental)

*Type location*: Lake Biwa, Shiga Prefecture, Japan

*Site of infection*: Myofibres in skeletal muscle of the prawn and in skeletal and cardiac muscles of the trout.

*Incidence*: Three out of 6 opaque *P. paucidens* among approximately 5000 collected individuals.

*Materials deposited*: Slides of a semi-thin section stained with 0.5% toluidine blue (MPM Coll. No. 21860-a for the muscle tissue of *O. mykiss* and -b for *P. paucidens*) and the ethanol-fixed muscle tissue of *O. mykiss* (21860-c) and *P. paucidens* (21860-d) were deposited at the Meguro Parasite Museum, Tokyo, Japan. All the materials were prepared from the specimens infected with *I. fujiokai* n. sp.

*Molecular characterization*: Nucleotide sequence of the SSU rDNA of *I. fujiokai* n. sp. was deposited in INSDC database with the accession number LC704895.

*Etymology*: The species name is dedicated to Dr Yasuhito Fujioka who for the first time confirmed the microsporidian disease in *O. mykiss* fed with *P. paucidens* and suggested the involvement of the prawn in the transmission in 1982.

## Data Availability

All the nucleotide sequences obtained in this study are available in INSDC database under the accession numbers LC704895–LC704899.
